# Ultraviolet Hyperspectral Interferometric Microscopy

**DOI:** 10.1038/s41598-018-28208-0

**Published:** 2018-07-02

**Authors:** Ashkan Ojaghi, Meredith E. Fay, Wilbur A. Lam, Francisco E. Robles

**Affiliations:** 1 Wallace H. Coulter Department of Biomedical Engineering, Georgia Institute of Technology and Emory University, Atlanta, Georgia USA; 2 Department of Pediatrics, Division of Pediatric Hematology/Oncology, Aflac Cancer Center and Blood Disorders Service of Children’s Healthcare of Atlanta, Emory University School of Medicine, Atlanta, Georgia USA

## Abstract

Ultraviolet (UV) spectroscopy is a powerful tool for quantitative (bio)chemical analysis, but its application to molecular imaging and microscopy has been limited. Here we introduce ultraviolet hyperspectral interferometric (UHI) microscopy, which leverages coherent detection of optical fields to overcome significant challenges associated with UV spectroscopy when applied to molecular imaging. We demonstrate that this method enables quantitative spectral analysis of important endogenous biomolecules with subcellular spatial resolution and sensitivity to nanometer-scaled structures for label-free molecular imaging of live cells.

## Introduction

The use of deep-UV light for microscopy offers many potential advantages over traditional methods, including higher spatial resolution due to the light’s shorter wavelength; and, when combined with spectroscopy, quantitative information with access to many endogenous molecules that play an important role in cell function and structure^1^. Unfortunately, there have been a number of challenges that have hampered progress in this area, including (1) less than optimal cameras and light sources, (2) phototoxicity, (3) strong fluorescent background, and (4) severe chromatic aberration^2^. But over the last decade, progress has been made to address some of these limitations^3–5^. For example, Zeskind *et al*. demonstrated that phototoxicity depends on exposure levels, and showed that live cells could be imaged contiguously with 280 nm light for over 6 hours before inducing visible damage^3^. Moreover, technological advances in sensors have led to the production of fast cameras with high dynamic range and high quantum efficiency in the UV; plus, broadband light sources in this spectral region have vastly improved.

UHI microscopy leverages these recent advances along with coherent detection to overcome all of the aforementioned limitations of molecular imaging in the deep-UV region of the spectrum. A key element of this technology is the use of a 4f interferometric setup that permits coherent detection using an incoherent, broadband light source^6,7^. The system consists of a modified Mach-Zehnder interferometer with a series of lenses in a 4f configuration to limit the number of modes illuminating each detected region of the sample, where we consider each light-mode to be given by the square of the transverse coherence length of the light source. With Fourier domain (FD) detection (i.e., light is detected as a function of wavelength), this configuration yields an interferometric signal with high fringe visibility, and access to the complex optical fields across a large spectral region (e.g., 240 nm–450 nm). In turn, this complex information allows us to correct for the chromatic aberration in the system by digitally refocusing. Further, coherent detection inherently eliminates the masking effects of fluorescence, an incoherent phenomenon that typically contaminates UV absorption images.

UHI microscopy also offers unique capabilities not seen in conventional microscopy. Specifically, the interferometric configuration makes it possible to extract quantitative spectroscopic information of both the absorptive and dispersive properties of many endogenous biomolecules that interact with light in the deep-UV region of the spectrum. (Dispersion refers to changes in the refractive index (RI) as a function of wavelength.) Moreover, this versatile tool can recover quantitative phase images (QPI)^8^ with sensitivity to optical path lengths in the order of 1 nm. Ultimately, UHI microscopy enables deep-UV, wideband, high-resolution spectroscopic measurements, with high spatial resolution and sensitivity to nanometer-scaled spatial fluctuations.

## Results

### UHI microscopy of a live red blood cell (RBC)

**Figure 1:**
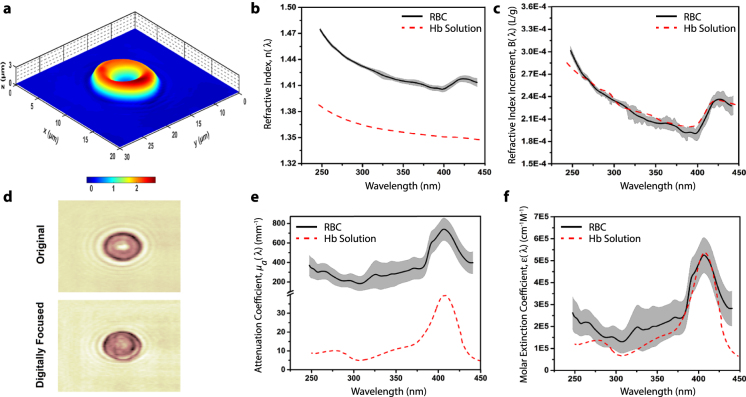
UHI microscopy of a live RBC. (**a**) Topological map of estimated thickness for an isolated human RBC (axes in μm). (**b**) Refractive index for RBC (black line) within one standard deviation range (gray area) and 2 g/dL Hb solution (dashed red line). (**c**) Real refractive index increment (B) spectra for the imaged RBC and Hb solution. (**d**) Example amplitude map of an imaged RBC before and after digital refocusing at 390 nm. Note that digital refocusing is applied to images at all wavelengths. (**e**) Attenuation coefficient spectra for RBC (black line) within its standard deviation range (gray area) and Hb solution (dashed red line). (**f**) Molar extinction coefficient spectra for the RBC and Hb solution.

The UHI microscopy system is described in the methods section and illustrated in Supplementary Fig. S1. To demonstrate the capabilities of UHI microscopy, we first present imaging results from human red blood cells, obtained from a whole blood sample of a healthy donor (methods section). RBCs represent an ideal test sample here since their hemoglobin (Hb) content can be considered homogeneous, and their shape and spectroscopic properties have been well characterized. Figure 1(a) clearly shows the RBC’s typical biconcave shape as determined by the quantitative phase imaging capabilities of UHI microscopy. The ability to overcome chromatic aberration is illustrated in Fig. 1(d), which shows an example amplitude image at 390 nm before and after digital refocusing—this is a critical step (repeated for every wavelength) for recovering in-focus images across the large spectrum, and hence for quantitative spectral analysis (see methods section and Supplementary Fig. S2)^9^.

Next, we compute the average wavelength-dependent attenuation coefficient and RI of the RBC. To achieve this, we first estimate the cell thickness from the measured QPI map at 300 nm by assuming a RI difference between the RBC (*n*_*RBC*_) and medium (*n*_*m*_) of 0.06 at this wavelength^10^. Note that the measured phase (*ϕ*) is related to cell thickness (*t*) and RI (*n*), by

1 $$\varphi (\lambda )={\rm{\Delta }}OPL(\lambda )\cdot 2\pi /\lambda =({n}_{RBC}(\lambda )-{n}_{m}(\lambda ))\cdot t\cdot 2\pi /\lambda $$

where ${\rm{\Delta }}OPL$ is the optical path length difference. The measured amplitude of the interferometric signal, $\tilde{A}$, is related to thickness and the attenuation coefficient (*µ*_*a*_), by

2 $$OD(\lambda )=-ln(\mathop{A}\limits^{ \sim }/{\mathop{A}\limits^{ \sim }}_{0})=1/2\cdot {\mu }_{a-Hb}(\lambda )\cdot t$$

where OD is the optical density and the factor of ½ accounts for the fact that we detect the amplitude of the optical field, not the intensity. After removing spectral changes due to cell thickness at each pixel, and accounting for the other constant parameters, we average the spectral information across the entire cell. The results are presented in Fig. 1, where many characteristic spectral features of Hb can be observed: in the attenuation coefficient (Fig. 1(e)), the Soret peak is clearly visible around 400 nm, while the RI (Fig. 1(b)) shows the strong inverse wavelength-dependence along with the characteristic dispersive ‘S’-shape around the Soret peak region.

To validate the RBC results, we measure the spectroscopic properties of a Hb-solution phantom with a concentration of ${c}_{Hb}=2\,g/dL$. To facilitate this comparison, we compute the molar extinction coefficient $({\varepsilon }_{Hb}(\lambda )=\,{\mu }_{a-Hb}(\lambda )/{c}_{Hb})$ for the RBC and the Hb-solution phantom, where we estimate the RBC’s Hb concentration to be ${c}_{Hb}=33.6\,g/dL$ (see supplementary information). The results, presented in Fig. 1(f), show excellent agreement between the two measurements and the expected behavior^11^. To compare the wavelength-dependent changes in refractive index of the RBC and Hb-solution phantom, we calculate the concentration independent, real refractive index increment of Hb (*B*), defined by

3 $$n(\lambda )={n}_{m}(\lambda )+{c}_{Hb}B(\lambda )$$

where *n(λ*) is the RI of the RBC or Hb-solution phantom. Results, presented in Fig. 1(c), again show excellent agreement between the two measurements and are also consistent with recently modeled values of B^10^.

### UHI microscopy of a live neutrophil

**Figure 2:**
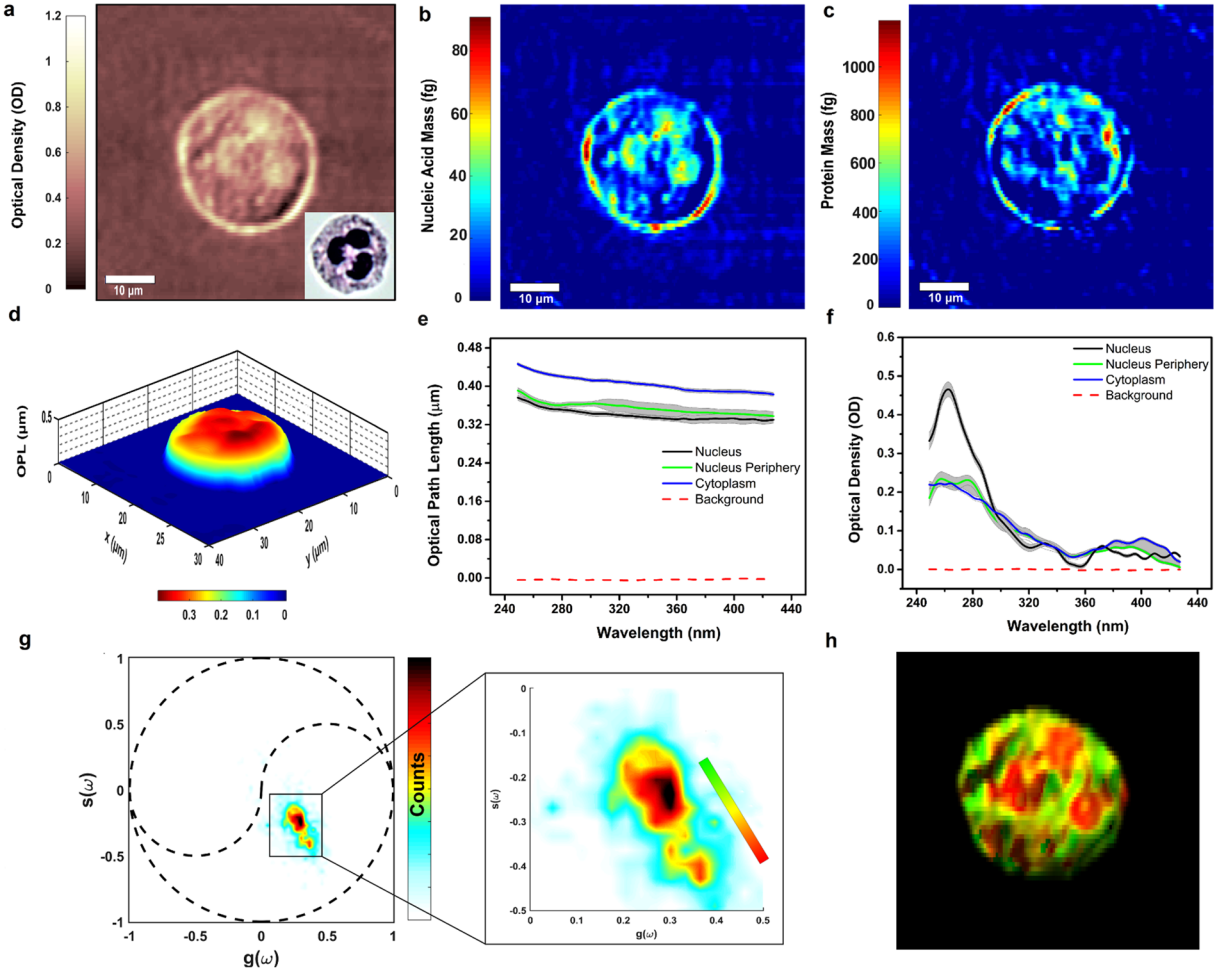
UHI microscopy of a live neutrophil. (**a**) Optical density (OD) map of an unstained, isolated human neutrophil at 260 nm wavelength with inset depicting a bright-field microscopy image of the same neutrophil after Giemsa staining. (**b**) Nucleic acid mass map of a neutrophil with an average mass of 23.81 fg within the cell. (**c**) Protein mass map of a neutrophil with an average mass of 273.65 fg within the cell. (**d**) OPL map of a neutrophil at 260 nm. (**e**) OPL spectra averaged over cell nucleus, nucleus periphery, and cytoplasm. (**f**) Optical density spectra extracted from cell nucleus, nucleus periphery, and cytoplasm. (**g**) Phasor space representation of the attenuation spectra obtained from the imaged neutrophil. Dotted black lines outline the unit circle and universal semicircles. (**h**) Molecular image obtained by color coding the phasor space clusters based on the color bar shown in inset of (**g**).

To further illustrate the capabilities of UHI microscopy, we image neutrophils, which contain a multi-lobular nuclear structure that is unique to polymorphonuclear leukocytes (also known as granulocytes). For imaging, the cells are attached to N-Formylmethionine-leucyl-phenylalanine (fMLP) coated quartz slides (see methods section). The optical density map of an isolated neutrophil at 260 nm (Fig. 2(a)) bears some of the characteristic features of these types of cells due to the highly absorbing regions of tightly packed nucleic acids in the nucleus, which possess an absorption peak at this wavelength. It is interesting to note that the corresponding QPI map at 260 nm (depicted in Fig. 2(d)) does not reveal a similar structure, likely due to contributions to the optical path length from other intracellular components, including the protein-filled granules. However, if we cytospin the neutrophils (instead of attaching them), the nuclear features become much more apparent in the phase images (see Supplementary Fig. S3). We attribute this difference to the cytospin process that likely spreads out heavy, overlapping intracellular components. Next, we use estimates of the molar extinction coefficients of proteins and nucleic acids at 260 nm and 280 nm to quantify their mass (Fig. 2(b,c)). Here we use a narrow spectral region (2 nm), which substantially reduces error from underestimating the molar extinction coefficients, and hence mass, by up to c.a. 17% (on average) compared to a broader (10 nm) bandwidth as previously reported^3^ (see supplementary information). The resulting nucleic acid mass map clearly shows the lobes of the nucleus, while the protein mass map highlights regions in the periphery of the lobes and throughout the cytoplasm.

Attenuation and dispersion spectra from selected regions in the nucleus, its periphery, and the cytoplasm reveal interesting spectral features. (Here we do not use a priori information about the RI of the sample, and hence its thickness, thus spectral results are reported as OD and OPL.) The average spectra extracted from the nucleus (in Fig. 2(f)) depicts the expected attenuation profile for nucleic acids with a peak at 260 nm, while the protein-rich areas in the periphery of the nucleus yield an average spectrum with a subtle shoulder at 280 nm. In contrast, the selected average spectra from the cytoplasm region, contains a weak peak centered at 415 nm which may be attributed to the presence of myeloperoxidase, an enzyme abundant in neutrophils^12^. The dispersion spectra shown in Fig. 2(e) show fewer unique spectral features, although in the particular selected areas, we do observe a lower overall OPL in areas corresponding to the nucleus compared to its immediate surrounding areas and the cytoplasm, consistent with recent reports^13^. This trend is not consistent throughout the entire cell when it is attached to the microscope slide—in this case the spatial structures of the QPI images reveal a more complex behavior—but a lower OPL indeed characterizes the nuclear structures in the neutrophils when they are processed using cytospin (see Supplementary Fig. S3).

### Unsupervised spectral analysis

Spectral signatures throughout the entire image are analyzed without a priori information using phasor analysis. In short, phasor analysis decomposes the spectral signals into two parameters, g and s, which are the projections of the spectra onto a cosine and sine function, respectively^14^. Here we use a frequency of 0.007 nm^−1^, corresponding to ~1¼ cycles from 240 nm to 450 nm. The distribution depicts two to three clusters which we color code with a continuous distribution ranging from red to green (Fig. 2(g)). The resulting molecular image in Fig. 2(h) clearly shows that the bottom cluster in phasor space (depicted in red in the molecular image) corresponds to the polymorphonuclear structure; while the middle region in phasor space (corresponding to yellow hues in the molecular image) overlaps mostly with the protein distribution around the nucleus. The upper cluster captures all other spectral signatures coming from the cytoplasm, and shows a broader distribution, which can be a result of spectral shifts, broadening, or the overlap of different molecules with varying properties. Surprisingly, phasor analysis for the wavelength-dependent OPL only reveals one cluster for these types of cells (Supplementary Fig. S4). This analysis highlights the diverse group of spectral signals that are acquired with UHI microscopy and that can be studied in an unsupervised fashion.

## Discussion and Conclusion

We have developed a versatile instrument that, to our knowledge, is the first to achieve hyperspectral imaging in the deep-UV, with access to attenuation and dispersion properties, and quantitative phase. The capabilities afforded by UHI microscopy overcome significant limitations that have plagued deep-UV microscopy, and offer new opportunities for highly-sensitive, label-free molecular imaging.

The ability to recover high-resolution biological images with absorption and dispersion information over a broad spectral range has only been demonstrated recently in the visible region of the spectrum (i.e., 450 nm–700 nm)^7,15–17^. Attempts to recover similar information in the deep UV, without imaging, have only covered a few select wavelengths, as the systems have required expensive, narrow-band, laser systems that have to be adjusted for each wavelength^18^. Consequently, quantitative phase imaging has only been demonstrated at a pair of UV wavelengths^19^. In contrast, UHI microscopy uses a modified interferometric system that enables coherent detection with an (inexpensive) incoherent, broadband UV light source to achieve hyperspectral imaging with access to many endogenous molecular species, without a priori information, and nanometer-scaled structural information.

In summary, UHI microscopy offers unique opportunities to leverage the biochemical specificity of UV spectroscopy for molecular imaging. This method has significant potential to provide detailed insight into biochemical and structural changes associated with, for example, disease progression or more dynamic processes like cell motility and proliferation, which will be explored in future work. We expect UHI microscopy to become an important tool for a variety of biomedical applications including characterization and phenotyping of cells and thin tissue biopsy samples.

## Methods

### UHI microscopy system design

The developed UHI microscopy system, depicted in Supplementary Fig. S1, consists of an incoherent broadband laser-driven plasma light source (EQ-99X LDLS, Energetiq Technology) along with a modified Mach-Zehnder interferometry configuration. The output light from the broadband source is collimated through a set of off-axis parabolic mirrors (Newport Corporation) and relayed via the 4f optical configuration. For imaging, we use 40X (NA 0.5) (LMU-40X, Thorlabs), UV microscope objectives and achieve an average spatial resolution of ~300 nm. Data are collected with an imaging spectrometer (IsoPlane-160, Princeton Instruments) equipped with a high-speed back-illuminated sCMOS camera (Kuro 1200, Princeton Instruments) (integration time is set to 100 ms). Information is recorded as a function of one spatial dimension (x-axis) along the rows of the camera, and spectrum across the columns of the camera. Then, the sample is translated across the orthogonal spatial dimension (y-axis) to recover a hyperspectral data cube. This is achieved via a high-precision motorized stage (MLS2031, Thorlabs). The total image acquisition time is dependent on the area of interest as scanning is required across one spatial dimension. The exposure of the camera (100 ms), limited by the light output of the source, is the rate limiting factor. An area covering 300 µm × 300 µm (750 lateral scans), for example, can be acquired in ~75 second. Future development of brighter, broadband UV light sources (with similar number of modes or less) can improve the imaging speed.

### Interferometric data analysis and digital refocusing

After acquiring an interferogram, which contains the signal as a function of wavelength and one spatial dimension (x-axis), we interpolate the data to wavenumber ($k=\frac{2\pi }{\lambda }$) and apply a 2-D Fast Fourier transform (FFT). The interferometric signal of interest is filtered (using a Butterworth filter) and shifted to DC to demodulate the interferometric signal, similar to holographic processing methods^20^. Then the inverse transform is taken which gives the complex field, i.e., the broadband amplitude and phase spectra of the field along one spatial dimension (x-axis). This process is repeated for each step along the y-axis to construct a 3D complex hyperspectral data cube. The measured complex field is used to mitigate the effects of chromatic aberration in the UV range. To this end, the complex field is refocused at all wavelengths using a Fresnel transformation^21^ where the wavefronts are digitally propagated according to the chromatic aberration-induced focal length shift of the UV objective (see Supplementary Fig. S2). This process yields focused amplitude and phase images throughout the imaging spectral range (e.g., 240 nm–450 nm). The phase information yields a resolution of 1.4 nm measured from the standard deviation of the phase map in a background region.

### RBC sample preparation

Whole blood was collected from healthy donors and added to an anticoagulant solution (sodium citrate, Beckton Dickenson) according to approved protocols by Institutional Review Board of Georgia Institute of Technology. Informed consent was obtained from healthy donors. Live red blood cells (RBCs) were isolated by centrifuging the whole blood sample at 150 g for 10 minutes, discarding the supernatant, washing with PBS at 400 g for 7 minutes twice, and resuspending in Phosphate-buffered saline (PBS) (Calbiochem). The cell suspension was pipetted into an imaging chamber placed on a quartz microscope slide and sealed with a quartz coverslip to avoid contamination and evaporation during the experiments.

### Hemoglobin solution sample preparation

Ferrous-stabilized human Hemoglobin (Sigma Aldrich) was added to PBS (Corning) to obtain a homogenous Hb solution of 2 g/dL concentration. The solution was then pipetted into sealed channels made using double-sided adhesive spacers (Electron Microscopy Sciences) attached between a quartz microscope slide and a quartz coverslip. This creates a layer of Hb solution with a thickness of approximately 133 μm.

### Neutrophil sample preparation

Live human neutrophils were isolated via negative magnetic antibody-based selection with the MACSxpress neutrophil isolation kit (Miltenyi Biotec) and resuspended in RPMI media with L-glutamine and HEPES (Life Technologies). In order to induce and maintain a normal cell spreading^22^ and adhesion to microscope slide surface, quartz slides were coated with a 1 nM solution of N-Formylmethionine-leucyl-phenylalanine (fMLP, Sigma-Aldrich) for 60 minutes, then rinsed with distilled water and PBS. The cell suspension was pipetted onto an imaging chamber placed on the coated slide and sealed with a quartz coverslip. In order to induce additional spreading on the neutrophils, cells were prepared on fMLP coated quartz slides using a Cytospin 4 (Thermo-scientific) at 500 g for 5 minutes.

### Neutrophil staining and microscopy

After imaging with UHI microscopy, neutrophils were stained using Giemsa and imaged using an upright (Axioskop 2 Plus, Carl Zeiss) microscope equipped with a 40x objective (numerical aperture (NA) 0.6), after performing UHI microscopy. The microscopy image of the stained neutrophils is available in Supplementary Fig. S5.

### Quantitative nucleic acid and protein mass mapping

We quantify the nucleic acid and protein mass based on calculation of the optical density ($OD(\lambda )=-\,ln(\tilde{A}/{\tilde{A}}_{0})$) at 260 nm and 280 nm wavelengths and assuming a linear contribution of species at each wavelength according to,

4 $$O{D}^{wavelengthn}=\frac{1}{2}({\varepsilon }_{nuc.\,acid}^{wavelength\,n})l{c}_{nuc.acid}+\frac{1}{2}({\varepsilon }_{protein}^{wavelength\,n})l{c}_{protein}$$

where ε is the extinction coefficient, $l$ is the path length, and c is the species concentration^3^. Again, note that we add a factor of ½ to account for the fact that we measure the amplitude of the optical field, not the intensity. Using OD values for the two wavelengths, we can generate two sets of equations and solve for the concentration-pathlength products ($lc$ terms) at each pixel. In our calculations we use average extinction coefficients at 260 nm and 280 nm for nucleic acid (${\varepsilon }_{260}=7000\,{M}^{-1}c{m}^{-1},{\varepsilon }_{280}=3500\,{M}^{-1}c{m}^{-1})$ and protein (${\varepsilon }_{260}=36,057\,{M}^{-1}c{m}^{-1},$ ${\varepsilon }_{280}=54,$ $129\,{M}^{-1}c{m}^{-1})$^3^ and average OD values over 2 nm and 10 nm bandwidths. Further, we assume an average molar mass of 52,728 Da for protein and of 330 Da for nucleic acid to obtain mass values at each pixel. The area in the sample being imaged onto one pixel in the camera is 0.1 µm^2^.

### Phasor analysis

Decomposition of spectra for each pixel into g and s parameters was performed using Eqs 5 and 6^14^.

5 $$g(\omega )=\frac{\int I(t)\cos (\omega t)dt}{\int |I(t)|dt}$$

6 $$s(\omega )=\frac{\int I(t)\sin (\omega t)dt}{\int |I(t)|dt}$$

The phasor plots of 5 cells are also shown in Supplementary Fig. S6. The average normalized attenuation spectra shown in Supplementary Fig. S6 were obtained by averaging the normalized spectra for all the pixels within a cluster defined by the hue in the molecular image.

## Electronic supplementary material

Supplementary Material
