# Supplementary material for: Ultraviolet Hyperspectral Interferometric Microscopy
Source: Sci Rep. 2018 Jul 2;8:9913. doi: 10.1038/s41598-018-28208-0 (PMC6028425; doi:10.1038/s41598-018-28208-0)
Supplement: Supplementary file 1 — Supplementary Material [file 41598_2018_28208_MOESM1_ESM.pdf]

# Supplementary Material for Ultraviolet Hyperspectral Interferometric Microscopy

Ashkan Ojaghi[1], Meredith E. Fay[1], Wilbur A. Lam[1], Francisco E. Robles[1,*]

[1] Wallace H. Coulter Department of Biomedical Engineering, Georgia Institute of Technology and Emory University, Atlanta, Georgia, USA.

*Correspondence should be addressed to F.E.R. (francisco.robles@bme.gatech.edu).

## Supplementary Information

## RBC Hb concentration estimation

In order to estimate the Hb concentration, we first calculate the attenuation coefficient ($\mu$) using our estimate of the cell thickness from the quantitative phase information. This allows us to isolate $\mu_{Hb}$ from the measured optical density, given by $OD(\lambda) = -ln(\tilde{A}/\tilde{A}_0) = ½ \cdot \mu_{Hb}(\lambda) \cdot t$. Since the wavelength-dependent attenuation coefficient ($\mu_{Hb}(\lambda)$) is linearly dependent on concentration and molar extinction coefficient ($\mu_{Hb}(\lambda) = c_{Hb}\varepsilon_{Hb}(\lambda)$), we can estimate the $c_{Hb}$ by using the molar extinction coefficient data compiled by Prahl[2] at a given wavelength (here $\lambda$ = 407 nm). Our estimation yielded a Hb concentration of 33.6 g/dL for the imaged RBC which is within the physiological range for healthy RBCs[3].

## Effect of wavelength bandwidth on protein and nucleic acid mass mapping

Although deep-UV microscopy has provided great potential for quantitative mass mapping of live cells, it only provides an estimation for the biomolecular mass distribution as the absorption images are inherently averaged over the bandwidth of UV filters used for imaging (10 nm in Zeskind et al.[4]). Looking into this issue, we repeated our mass mapping procedure using average attenuation maps over a 10 nm bandwidth at 260 nm and 280 nm wavelengths. The results (shown in supplementary Fig. S7) indicate an expected underestimation for both nucleic acid and protein mass of 5.3% and 17.4 % (averaged across the entire cell), respectively, when using a filter with a wide-bandwidth. This underestimation can be avoided with UHI microscopy given its flexibility to extract only a narrow spectral range.

## Phase unwrapping for QPI

Use of short wavelengths for QPI can be problematic given that small changes in optical path length can lead to multiple 2π-jumps that produce phase-wrapping artifacts. However, UHI microscopy overcomes this limitation by leveraging its spectral content which permits use of robust frequency-estimation-based unwrapping methods[5]. First, the phase information is inherently oversampled in the spectral domain, thus no abrupt phase jumps are expected. Secondly, the phase spectrum at each pixel can be is unwrapped and fitted to a line of the form of $\phi(\lambda) = \frac{2\pi}{\lambda} OPL' + 2\pi \cdot m$. Here $OPL'$ is the best estimate of the optical path length and $m$ yields the integer 2π-jumps. Thus, the spectral information makes UHI less susceptible to phase wrapping artifacts.

## Supplementary Figures

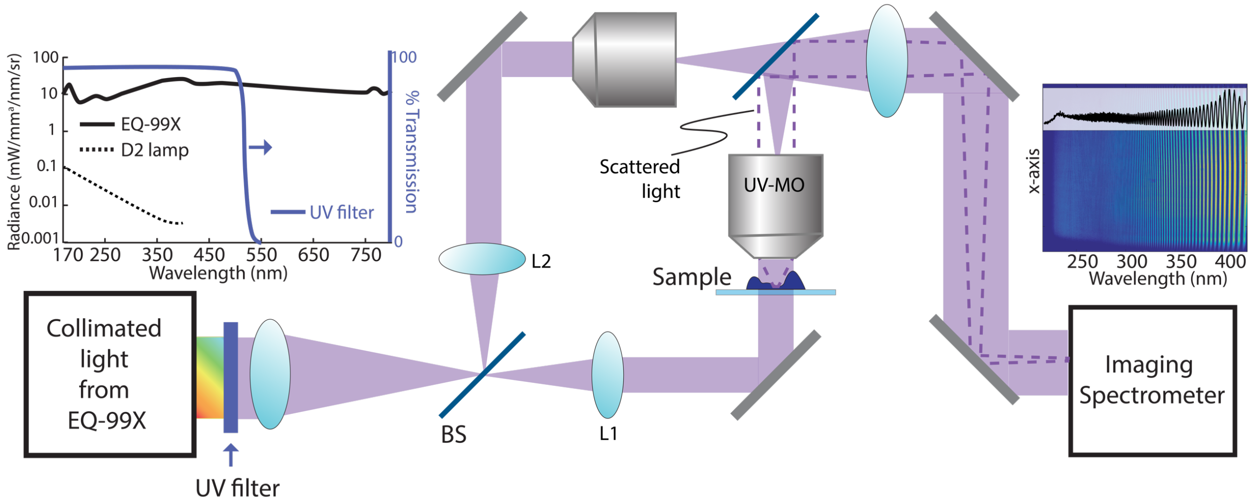


**Figure S1. Design of the UHI microscopy system**. Schematic of the designed UHI microscopy setup where the collimated broadband beam (radiance spectrum of the source output light is depicted in the left inset). The beam is split into reference and sample beams via the first beam splitter (BS) and collimated using L1 and L2 lenses. The imaging is performed using UV microscope objective (UV-MO) and the interferometric data (a sample image is shown in the right inset) is recorded by the imaging spectrometer.

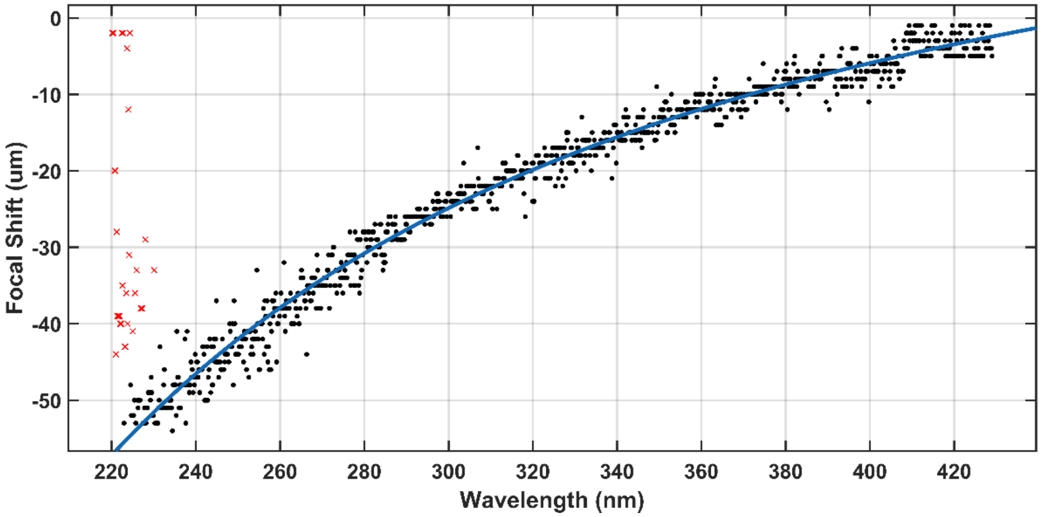


**Figure S2. Aberration-induced focal length shift of the UV objective**. Plot showing the focal length shift for the UV objective used in UHI microscopy setup throughout the imaging wavelength range obtained via quantitative characterization of the focal shift based on minimum image entropy focus detection method[1].

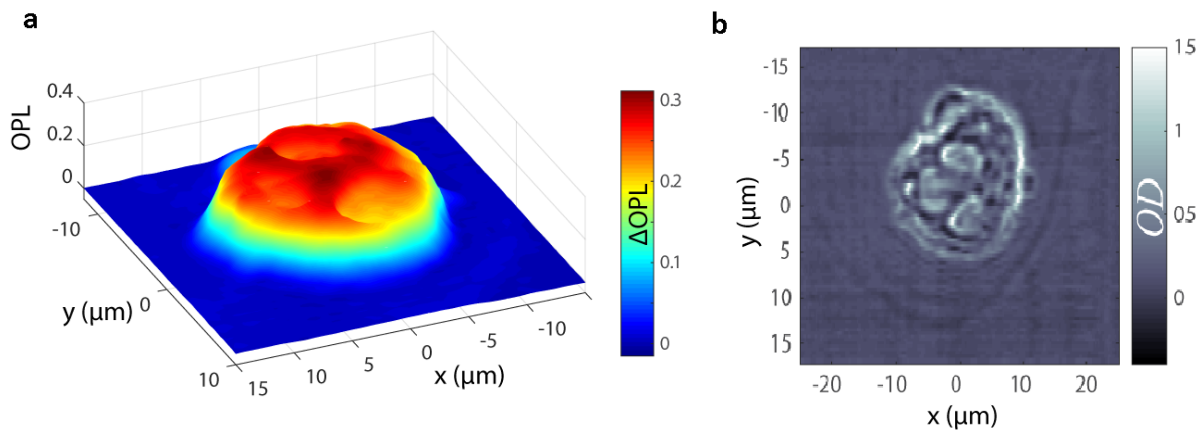


**Figure S3. OPL and OD maps at 260 nm of a neutrophil prepared via cytospin**. (a) three-dimensional OPL map obtained by cytospinning the neutrophils on a fMLP coated quartz slide, showing lower OPL for the lobular structure of the cell nucleus. (b) OD map of the imaged cytospun neutrophil indicating a multi-lobular structure for the cell nucleus with higher density compared to the cytoplasm.

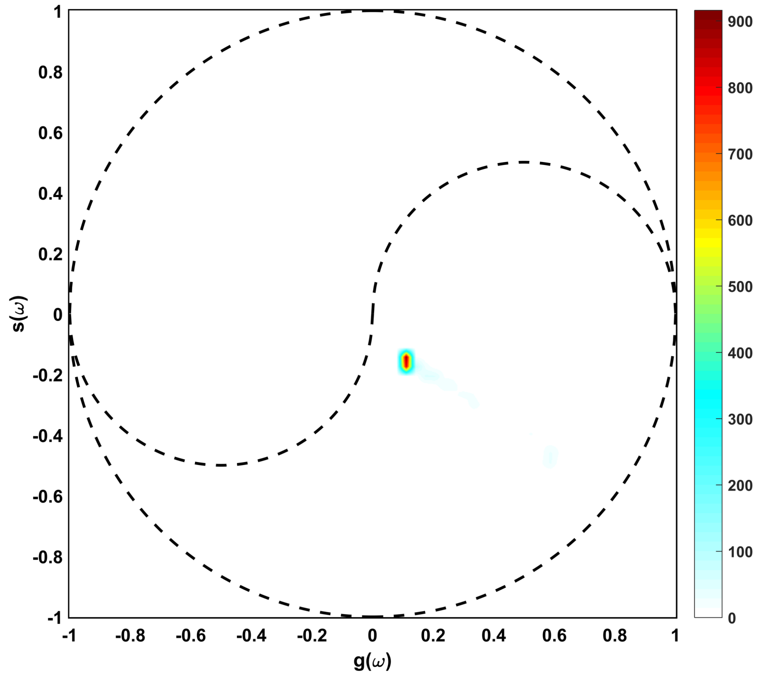


**Figure S4. OPL phasor space of a neutrophil**. The phasor space plot based on decomposition of OPL spectra showing only one dense cluster.

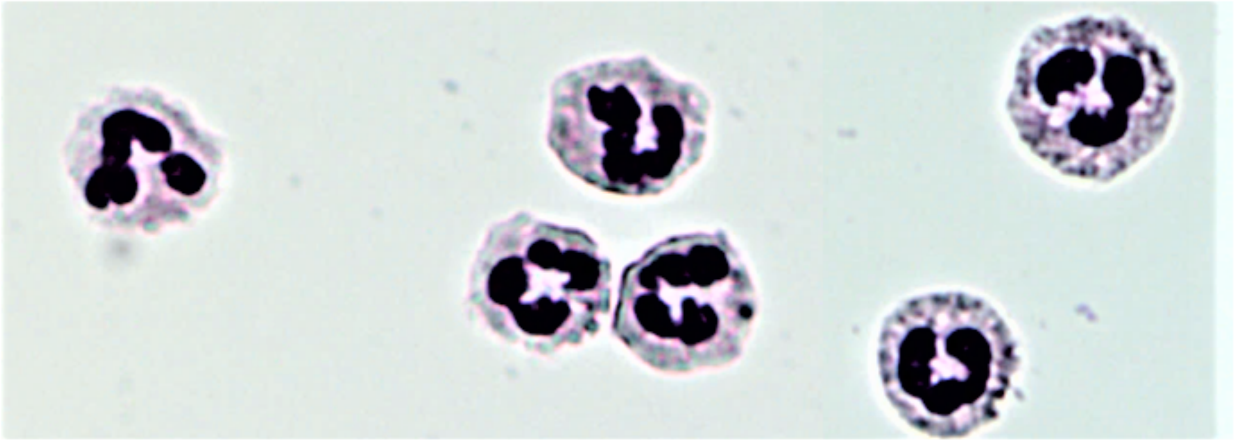

**Figure S5. Giemsa stained neutrophils**. Microscopy images of Giemsa stained neutrophils after UHI microscopy and imaged via a conventional upright microscope, showing the multi-lobular nuclear structure.

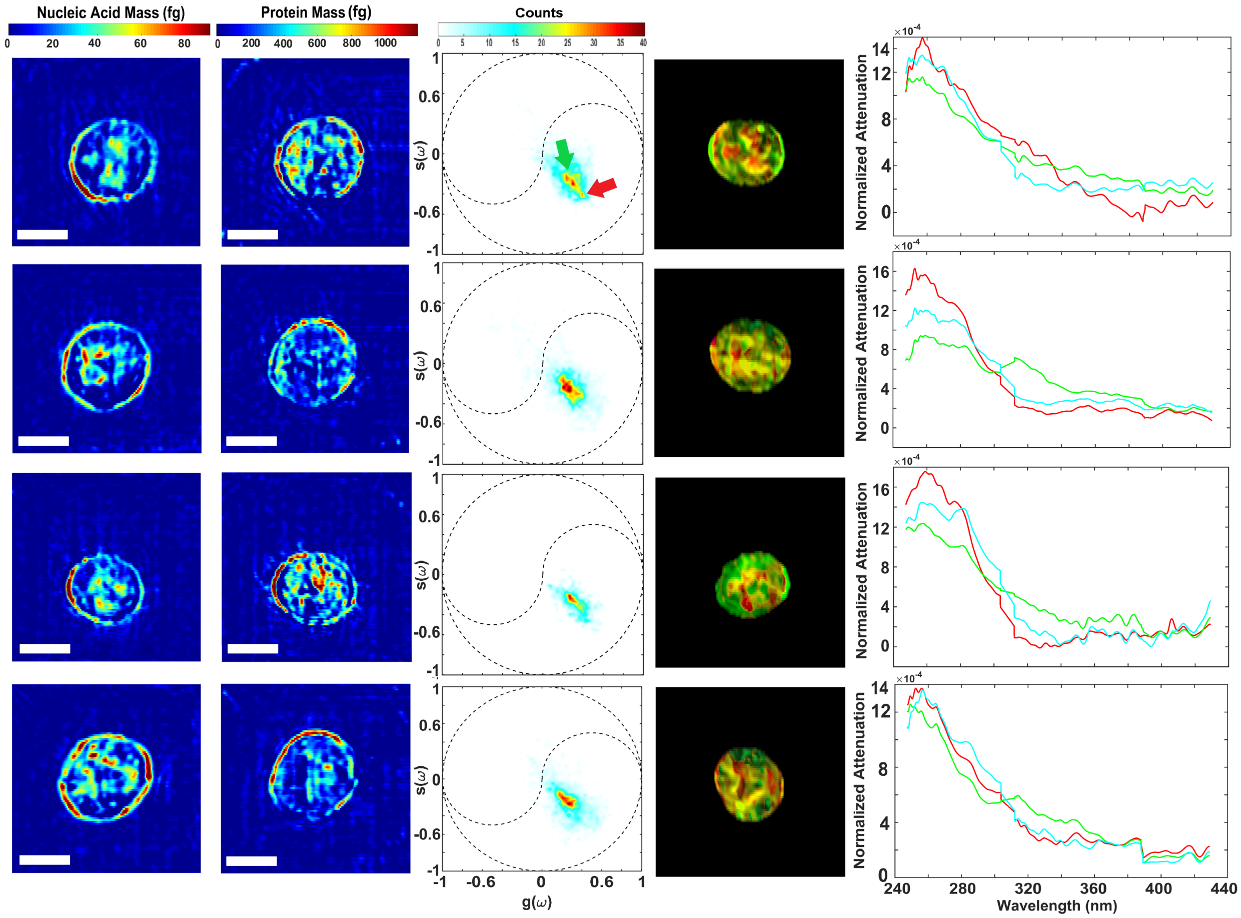


**Figure S6. Phasor analysis and quantitative mass mapping of four neutrophils**. Quantitative nucleic acid and protein mass maps (scale bars, 10 μm), phasor space representations (arrows indicate the color-coding scheme used), color-coded molecular images, and average normalized attenuation spectra for clusters color-coded in the molecular image by red, green and yellow hue (spectra plotted in cyan for better visibility).

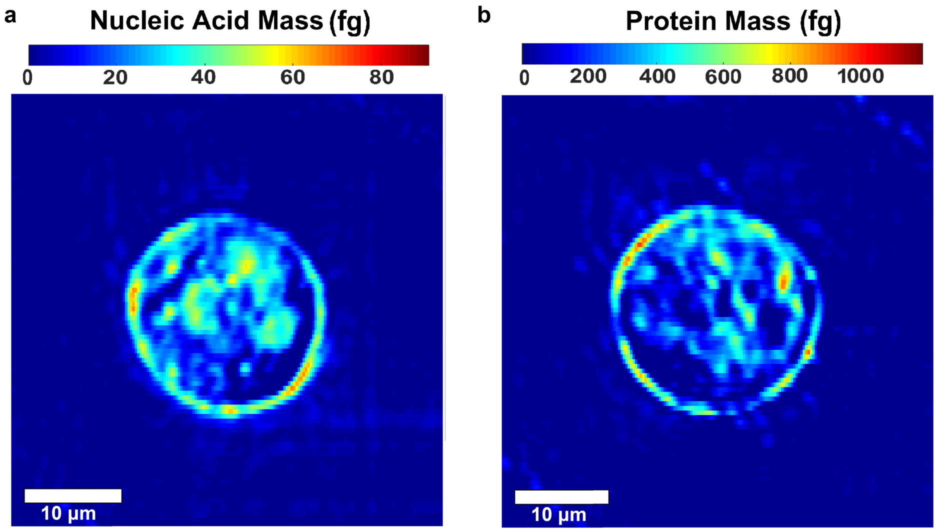


**Figure S7. Underestimated protein and nucleic acid mass in UV imaging**. Nucleic acid (a) and protein mass (b) maps obtained using OD maps averaged over 10 nm bandwidth, showing underestimation of both nucleic acid (5.3% average error) and protein (17.4% average error) mass.

**References:**


1. Gillespie, J. & King, R. A. The use of self-entropy as a focus measure in digital holography. *Pattern Recognit. Lett.* **9,** 19–25 (1989).

2. Prahl, S. Optical absorption of hemoglobin. *http//omlc. ogi. edu/spectra/hemoglobin* (1999).

3. Robles, F. E., Samineni, P., Wilson, J. W. & Warren, W. S. Pump-probe nonlinear phase dispersion spectroscopy. *Opt. Express* (2013). doi:10.1364/OE.21.009353

4. Zeskind, B. J. *et al.* Nucleic acid and protein mass mapping by live-cell deep-ultraviolet microscopy. *Nat. Methods* **4,** 567–569 (2007).

5. Shen, F. & Wang, A. Frequency-estimation-based signal-processing algorithm for white-light optical fiber Fabry–Perot interferometers. *Appl. Opt.* **44,** 5206 (2005).
